# Seroprevalence of toxoplasmosis, rubella, cytomegalovirus, herpes simplex, and syphilis infections among pregnant women and infants in Malaysia: a systematic review and meta-analysis

**DOI:** 10.7717/peerj.21632

**Published:** 2026-08-18

**Authors:** Abdirahman Hussein Elmi, Ahmad Adebayo Irekeola, Hafeez Abiola Afolabi, Khalid Hajissa, Yusuf Wada, Chan Yean Yean, Zeehaida Mohamed

**Affiliations:** 1 Department of Medical Microbiology and Parasitology, School of Medical Sciences, Health Campus, Universiti Sains Malaysia, Kubang Kerian, Malaysia; 2 Department of Medical Laboratory Sciences, Faculty of Medicine and Health Sciences, Jamhuriya University of Science and Technology, Mogadishu, Somalia; 3 Basic Sciences Unit, School of Dental Sciences, Health Campus, Universiti Sains Malaysia, Kubang Kerian, Malaysia; 4 Microbiology Unit, Department of Biological Sciences, College of Natural and Applied Sciences, Summit University Offa, Offa, Kwara State, Nigeria; 5 Department of General Surgery, School of Medical Sciences, Hospital Universiti Sains Malaysia, Health Campus, Universiti Sains Malaysia, Kubang Kerian, Malaysia; 6 Department of Zoology, Faculty of Science and Technology, Omdurman Islamic University, Omdurman, Sudan; 7 Department of Zoology, Faculty of Life Sciences, Ahmadu Bello University, Zaria, Nigeria; 8 Hospital Pakar Universiti Sains Malaysia, Kubang Kerian, Malaysia

**Keywords:** Seroprevalence, TORCH infection, Pregnant women, Infant, Meta-analysis, Malaysia

## Abstract

**Background:**

Toxoplasmosis, rubella, cytomegalovirus, herpes simplex, and syphilis (TORCH) infections in infants and pregnant women are a cause for concern. However, the true prevalence of the infections among these populations in Malaysia is unknown.

**Methodology:**

This study represents the first systematic review and meta-analysis to assess the seroprevalence of TORCH agents in pregnant women and infants in Malaysia. Data from scientific articles published until November 2025 were analyzed.

**Results:**

A total of 19 reports on the seroprevalence of infection by TORCH agents in Malaysia were analyzed. The overall seroprevalence was 32.5% (95% CI [16.6–53.8%]; *I*^2^ = 99.4%; *p* < 0.001) among pregnant women and infants in Malaysia. Based on the target population, 27.7% and 35.1% seroprevalence were found in pregnant women and infants, respectively. Among the TORCH agent types, Cytomegalovirus (CMV) in pregnant women and infants demonstrated the highest seroprevalence (93.2% and 95.9%, respectively), while *Treponema pallidum* (*T. pallidum*) seroprevalence was the lowest among pregnant women (1.1%) and infants (4%).

**Conclusions:**

This study reveals a high seroprevalence of TORCH infection and underscores the importance of adequately screening pregnant women to prevent potential vertical transmission to the neonate.

## Introduction

TORCH refers to a specific group of pathogens including *Toxoplasma gondii* (*T. gondii*), other agents such as *Treponema pallidum* (*T. pallidum*), Rubella virus, Cytomegalovirus (CMV), and Herpes simplex virus (HSV) that pose a substantial threat to global maternal and fetal health (Batra, Batra & Singh, 2020). Globally, millions of pregnancies are complicated by these pathogens annually, disproportionately contributing to the burden of adverse neonatal outcomes, including stillbirths, premature delivery, and severe congenital anomalies (Batra, Batra & Singh, 2020; Gouda et al., 2023). Because these infectious agents possess the unique ability to breach the placental barrier following systemic maternal infection, they can directly invade fetal tissues *via* transplacental transmission. This vertical transmission can result in permanent sequelae, including microcephaly, sensorineural hearing loss, congenital heart defects, and severe neurodevelopmental delays (Mohamed & Hajissa, 2016; Thangarajah et al., 2019; Gouda et al., 2023). Consequently, the sheer magnitude of this disease burden underscores the critical necessity for routine prenatal screening and early therapeutic intervention to reduce neonatal morbidity and mortality (Kamel, Barakat & Abbas, 2024; Patel et al., 2024).

Despite the recognized clinical severity of these infections, navigating maternal screening on a global scale remains highly complex. While high-income nations have successfully integrated standardized TORCH screening and vaccination programs to drastically reduce transmission, these pathogens remain heavily prevalent in low- and middle-income countries (Menendez et al., 2017; Rajendiran et al., 2021). In these transitioning regions, limited access to early diagnostic tools and comprehensive prenatal services allows these infections to propagate undetected (Menendez et al., 2017; Rajendiran et al., 2021). This public health challenge is further compounded by the insidious clinical nature of the pathogens themselves. Because initial maternal infections often lack symptoms, infected newborns frequently show no obvious clinical signs at birth. This absence of visible indicators severely delays diagnosis, causing infants to miss critical windows for early therapeutic intervention (Morsy, Hussein & Morsy, 2022).

Compounding these global epidemiological challenges are the inherent limitations of standard diagnostic modalities. Traditional serological assays, which remain the primary screening tools globally, present significant interpretative hurdles even within resource-replete clinical settings (Chung, Shin & Lee, 2018). A fundamental diagnostic challenge lies in differentiating an acute, active infection, which poses an immediate threat to the fetus, from historical exposure or acquired immunity. Maternal immunoglobulin G (IgG) antibodies may persist for several years, and IgG passively acquired *via* transplacental transfer can severely complicate neonatal assessments (Chen et al., 2019; Batra, Batra & Singh, 2020; Fuchs et al., 2021; Deka et al., 2022). Furthermore, although the presence of immunoglobulin M (IgM) is typically indicative of a recent primary infection, its diagnostic utility is frequently constrained by cross-reactivity with heterologous pathogens, leading to potential false-positive results (Chen et al., 2019; Batra, Batra & Singh, 2020; Fuchs et al., 2021; Deka et al., 2022). Because the accurate interpretation of these complex serological profiles relies heavily on understanding the baseline immunity of a given population, establishing robust, localized seroprevalence data is a critical scientific prerequisite (Batra, Batra & Singh, 2020; Fuchs et al., 2021; Deka et al., 2022; Abu Shqara et al., 2025).

While Malaysia seeks to enhance maternal and child health outcomes, a comprehensive understanding of the true frequency of TORCH infections is currently hindered by the absence of routine, nationwide prenatal screening. Despite Malaysia’s relatively robust healthcare infrastructure, routine TORCH screening remains largely unstandardized, primarily because policymakers lack consolidated baseline data to evaluate its necessity and cost-effectiveness (Andiappan et al., 2014a; Yeoh, Hornetz & Dahlui, 2016; Hamid et al., 2022; Abu Bakar et al., 2025). Existing data are fragmented, limited to small regions or individual pathogens, and show significant variations in seroprevalence rates (Fuchs et al., 2021; Abu Bakar et al., 2025). Furthermore, Malaysia presents a unique epidemiological landscape characterized by a tropical climate, a diverse multi-ethnic population, and a high influx of migrant workers from neighboring endemic regions, which may significantly influence exposure risks and immunity levels (Fuchs et al., 2021; Abu Bakar et al., 2025). Addressing these specific epidemiological and clinical gaps is essential.

The primary aim of this study is to determine the seropositivity of TORCH infections among Malaysian pregnant women and infants to ascertain the actual epidemiological situation. Since there is currently no published meta-analysis on this subject, the authors performed a thorough systematic review to provide a unified, robust dataset. By synthesizing this fragmented data, this review specifically intends to bridge the current policy gap by providing the evidence base required for health authorities to re-evaluate prenatal screening guidelines. Ultimately, defining the disease burden in Malaysia will not only inform localized public health interventions but also serve as a valuable reference for navigating TORCH screening policies in similarly transitioning middle-income countries in the region (Fuchs et al., 2021).

## Materials and Methods

This systematic review and meta-analysis of various types of research and published articles was conducted in accordance with the Preferred Reporting Items for Systematic Reviews and Meta-Analysis (PRISMA) guidelines (Moher et al., 2009), as shown in the File S1. The study procedure/protocol was registered with PROSPERO (registration number: CRD42022377279).

### Literature search strategy

In this study, several published articles were retrieved from four major electronic databases (Web of Science, Google Scholar, Scopus and PubMed). To determine whether the research objective had been achieved, the eligible studies were searched and screened using comprehensive and related keywords: (Toxoplasma OR toxoplasmosis OR Syphilis OR “Rubella virus” OR Rubella OR Cytomegalovirus OR CMV OR “Herpes simplex virus” OR HSV OR HSV-1 OR HSV-2 AND “Malaysia”). The Supplemental search strategy file (File S2) contains a complete list of the search strategies used in this study. The databases were last searched on November 28, 2025.

All retrieved records from the databases searched were exported to EndNote X8 for aggregation. The duplicate removal workflow was conducted first, utilizing EndNote’s automated find duplicates function, followed by a thorough manual verification by the reviewing team to ensure no duplicates were missed. The unique articles were carefully assessed for eligibility by two independent reviewers (AHE and ZM), and the results were reviewed by the third reviewer (KH). The titles and abstracts of the articles were firstly perused, followed by full-text evaluation when a decision cannot be reached based on title and abstract assessment alone. Discrepancies were resolved by discussion among the reviewers.

### Eligibility criteria

Studies with designs including retrospective, cross-sectional, prospective cohort, and randomized clinical trials were included. Original studies were only included if they provided details on the seroprevalence of TORCH infections, specifically reporting serological data such as the detection of IgG and/or IgM antibodies among pregnant women and infants (under one year old) in Malaysia. Studies based only on clinical symptoms, non-serological investigations, case reports, comments, editorials, reviews, or non-human subjects were excluded. All studies with insufficient information and studies whose full text could not be retrieved were also excluded. When a single paper reported data for multiple pathogens, each pathogen was extracted as a separate study unit.

### Data extraction

The data were extracted using an Excel spreadsheet. The titles and abstracts were independently verified by three reviewers (Abdirahman Hussein Elmi, Zeehaida Mohamed, and Khalid Hajissa). Principal data on the number of participants examined for anti-TORCH antibodies among pregnant women and infants in Malaysia, along with the number of seropositive cases, were extracted from all eligible articles. The following details were also documented: author and publication year, study period, target population, age range, location, sample size, TORCH agent, and detection method.

### Quality assessment

Two authors independently evaluated the methodological integrity of each included study using the critical appraisal tool established by Joanna Brigg’s Institute (JBI) for prevalence investigations (File S3). Furthermore, the evaluations were validated by another independent author, and any significant discrepancies were addressed *via* verification and discussion. The quality assessment tool has nine questions, each with a “yes”, ”no”, “unclear”, or ”not relevant” response. The percentage of “yes” responses to the items was used to score all studies. A high-quality study has a high proportion of “yes” (70% and above), while 50%–69% and 49% or below correlate to moderate- and low-quality studies (Munn et al., 2015; Irekeola et al., 2022). File S4 contains information on the quality of the 13 included studies.

The methodological quality of the included studies was evaluated using the Joanna Briggs Institute (JBI) critical appraisal checklist for prevalence studies. Two reviewers independently assessed each study based on sample frame adequacy, recruitment methods, sample size, study description, and data analysis. The evaluation focused on sample frame adequacy, recruitment methods, sample size justification, detailed subject descriptions, and data analysis rigor. Any disagreements between the reviewers were resolved by consensus or by a third reviewer. No studies were excluded based on quality scores alone, but the scores were used to evaluate the overall strength of the findings.

### Data synthesis and statistical analysis

Open Meta Analyst software and Comprehensive Meta-Analysis version 2 were used for the analysis of the data (Higgins & Thompson, 2002; Irekeola et al., 2022). The summary estimates of the seroprevalence of TORCH agents in pregnant women and neonates were computed, and subgroup analysis was performed based on the target population, location, TORCH agents, and detection method. Pooled estimates were determined using random effect model and the DerSimonian-Laird method of meta-analysis. Using funnel plot, small study effects or potential publication bias were evaluated. The asymmetry of the plot was assessed using Egger’s regression test. The heterogeneities of study-level estimates were assessed by Cochran’s Q test and quantified using *I*^2^ statistics. *I*^2^ values of 25%, 50%, and 75% were regarded as indicating low, moderate, and high heterogeneity, respectively (Higgins & Thompson, 2002). For all tests, *p* < 0.001 was considered significant. Subgroup analysis was systematically performed based on the target population, location, TORCH agents, and detection method to investigate sources of heterogeneity. Finally, small study effects or potential publication bias were evaluated using a funnel plot, and the asymmetry of the plot was formally assessed using Egger’s regression test.

## Results

**Figure 1:**
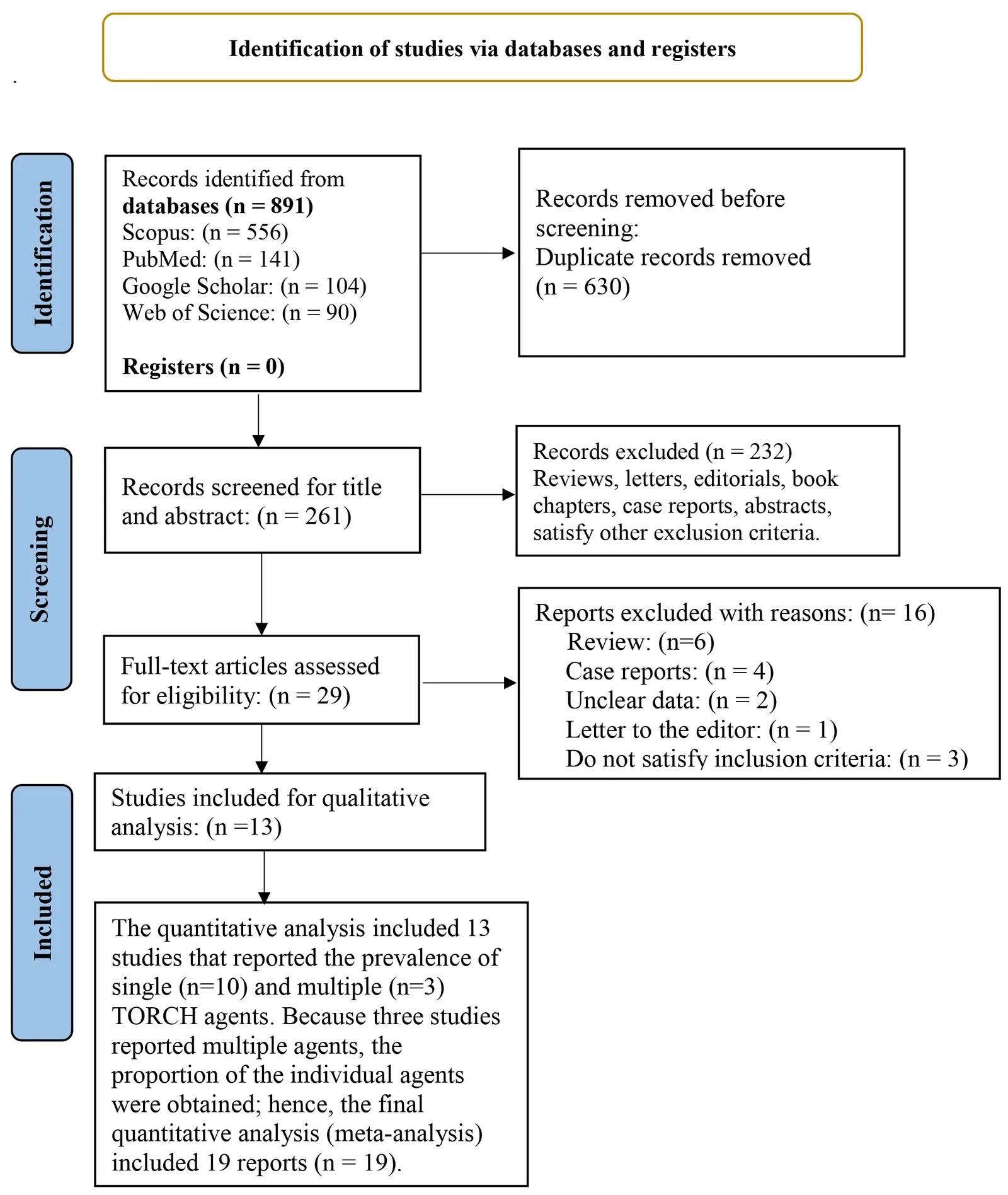
PRISMA flow diagram for the selection of eligible articles included in the study.

The initial literature search yielded a total of 891 records across all databases (PubMed (*n* = 141), Web of Science (*n* = 90), Scopus (*n* = 556), and Google Scholar (*n* = 104)). After removing 630 duplicates and screening 261 titles and abstracts, 232 articles were excluded. Ultimately, 13 full-text studies met the inclusion criteria (Tan & Stern, 1981; Tan, 1985; Goh & Ngeow, 1989; Balasubramaniam et al., 1994; Ai & Ee, 2008; Saraswathy et al., 2011; Andiappan et al., 2014b; Emelia et al., 2014; Mohamed & Hajissa, 2016; Ahmad et al., 2020; Hamid et al., 2022; Elmi et al., 2024b; Elmi et al., 2024a) (Fig. 1). To ensure a precise estimation of individual TORCH agents, data from studies reporting multiple agents were analyzed as separate reports. While ten studies focused on a single agent, three studies reported on multiple agents; thus, these were divided into their respective components, resulting in a final meta-analysis of 19 reports (Table 1).

**Figure 2:**
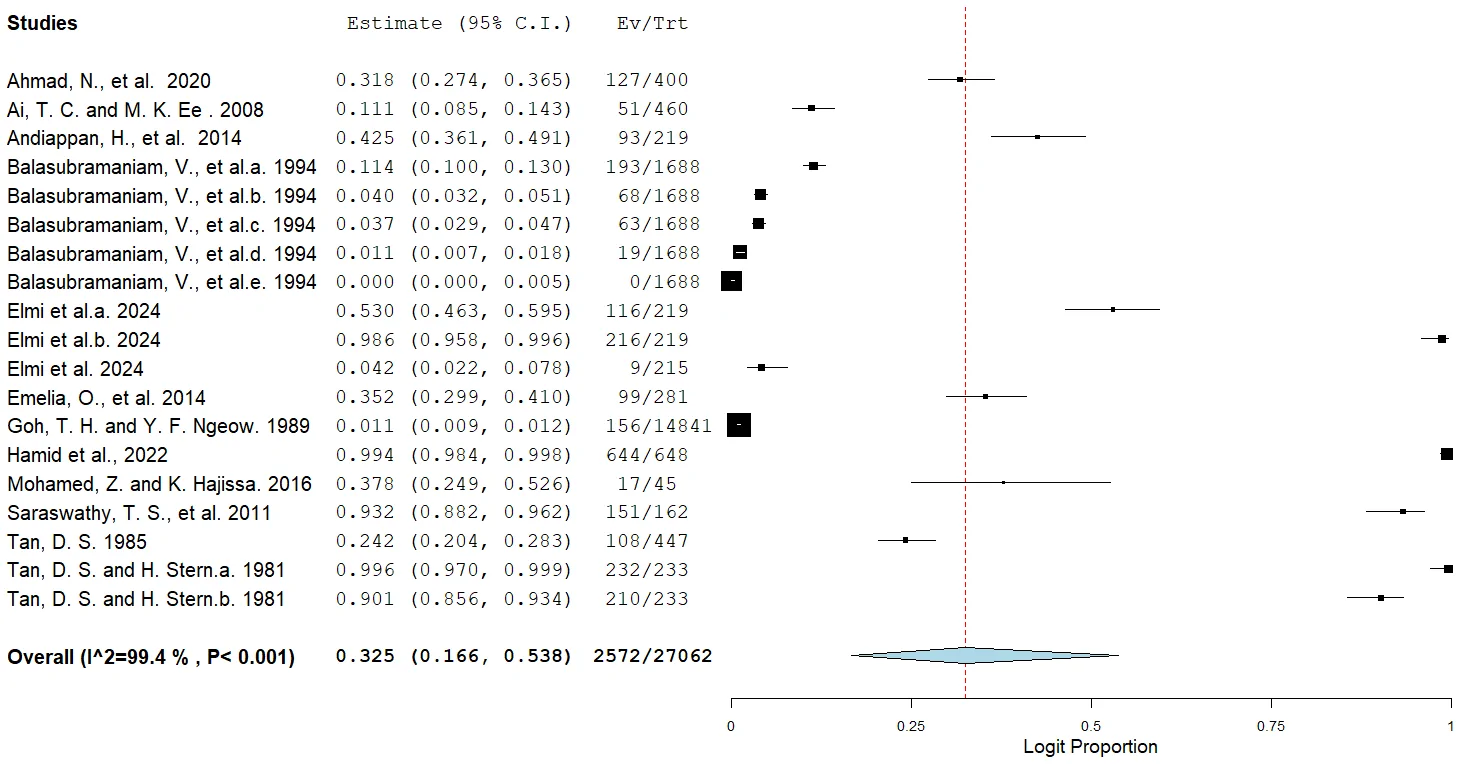
Overall seroprevalence of TORCH agents among pregnant women and infants.

**Figure 3:**
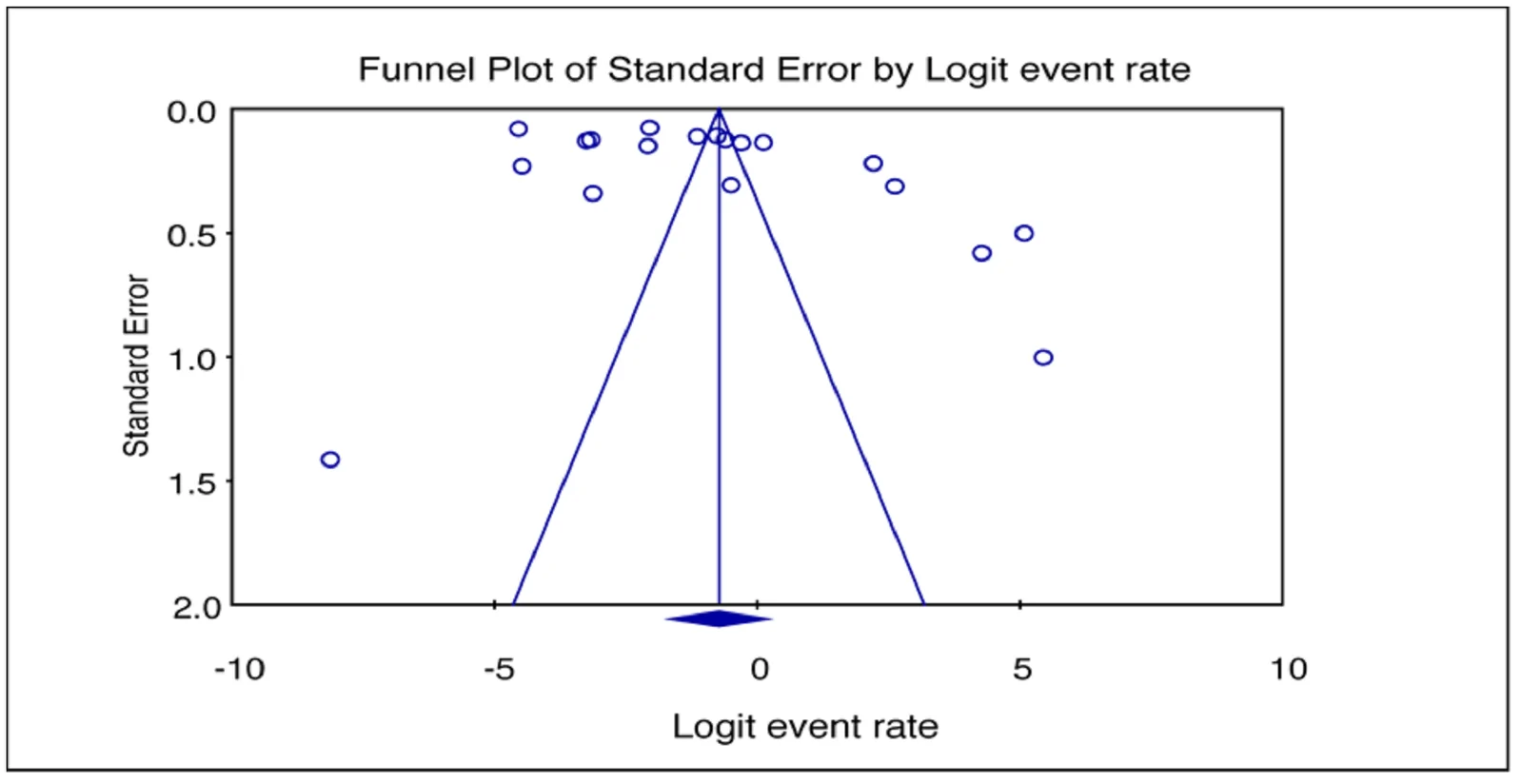
Funnel plot showing no significant evidence of publication bias (Egger’s test, *p* = 0.0.0752).

Overall seroprevalence of TORCH agents among pregnant women and infants Using a random effect meta-analysis, the overall seroprevalence of TORCH agents was estimated to be 32.5% (95% CI [16.6–53.8]%; *I*^2^ = 99.4%; *p* < 0.001) among pregnant women and infants in Malaysia (Fig. 2). No evidence of publication bias was observed upon generating a funnel plot of the included studies (Fig. 3). In addition, Egger’s regression test revealed a non-significant *p*-value (*p* = 0.0752) when the plot’s asymmetry was assessed.

**Table 1:** Characteristics of studies reporting the prevalence of TORCH agents in Malaysian pregnant women and infants.

**NO**	**Author,** **Publication year**	**Study period**	**Target population**	**Age range**	**Location**	**Sample size**	**Serology positive n (%)**	**TORCH agents**	**Detection method**
1	Ahmad et al. (2020)	February to May 2015	Pregnant women	≥20	Kuala Lumpur	400	127 (31.83)	*T. gondii*	ELISA
2	Ai & Ee (2008)	June to October2005	Pregnant women	16–45	Selangor	460	51 (11.08)	*Rubella virus*	MEIA
3	Andiappan et al. (2014b)	NR	Pregnant women	20–41	Kuala Lumpur	219	93 (42.47)	*T. gondii*	ELISA
4	Balasubramaniam et al. (1994)	1961–1979	Infants	0–4 MTHS	Multilocation	1,688	193 (11.4)	CMV	ELISA
5	Balasubramaniam et al. (1994)	NR	Infants	0–4 MTHS	Multilocation	1,688	68 (4)	*T. pallidum*	VDRL
6	Balasubramaniam et al. (1994)	NR	Infants	0–4 MTHS	Multilocation	1,688	63 (3.7)	*Rubella virus*	HI & HIT
7	Balasubramaniam et al. (1994)	NR	Infants	0–4 MTHS	Multilocation	1,688	19 (1.0)	*T. gondii*	IFA
8	Balasubramaniam et al. (1994)	NR	Infants	0–4 MTHS	Multilocation	1,688	0 (0)	HSV	CF
9	Elmi et al. (2024a)	January to December 2022	Newborn	<1 MTH	Kelantan	219	116 (53)	T. gondii	ECLIA
10	Elmi et al. (2024b)	January to December 2022	Newborn	<1 MTH	Kelantan	219	216 (98.6)	CMV	ECLIA
11	Elmi et al. (2024a); Elmi et al. (2024b)	January to December 2022	Newborn	<1 MTH	Kelantan	215	9 (4.2)	HSV	ECLIA
12	Emelia et al. (2014)	October 2012 and May 2013.	Pregnant women	NR	Kuala Lumpur	281	99 (35.23)	*T. gondii*	ELISA
13	Goh & Ngeow (1989)	NR	Pregnant women	NR	Kuala Lumpur	14,841	156 (1.05)	*T. pallidum*	VDRL & TPHA
14	Hamid et al., (2022)	January to December 2018	Infants	<1 MTH	Kelantan	648	644 (99.3)	CMV	ECLIA
15	Mohamed & Hajissa (2016)	NR	Newborn	<1 MTH	Kelantan	45	17 (37.77)	*T. gondii*	ELISA
16	Saraswathy et al. (2011)	January 2007– December 2008.	Pregnant women	NR	Multilocation	162	151 (93.21)	CMV	ELISA
17	Tan (1985)	NR	Infants	0–4 MTHS	Multilocation	447	108 (24.16)	*Rubella virus*	HI & HIT
18	Tan & Stern (1981)	1961–1979	Infants	0–11 MTHS	Multilocation	233	232 (99.57)	CMV	CF
19	Tan & Stern (1981)	1961–1979	Infants	0–11 MTHS	Multilocation	233	210 (90.13)	HSV	CF

**Notes.**

TORCH Toxoplasma, Others, Rubella, Cytomegalovirus, Herpes simplex virus CI confidence intervals cCMV congenital CMV ELISA Enzyme-linked immunosorbent Assay MEIA microparticle enzymes immunoassay VDRL Venereal Disease Research Laboratory HI Hemagglutination Inhibition HIT Hemadsorption Immunosorbent Technique CF Complement Fixation TPHA Treponema pallidum Hemagglutination Assay MTHS Months NR No report

### Subgroup meta-analysis

Subgroup analysis was also performed for four different parameters, namely: the target population of the included studies, the location where the surveys/studies were described, the type of TORCH agents, and the method employed in detection of seroprevalence of TORCH agents. This subgroup analysis will help to recognize the potential sources of heterogeneity among studies, as significant heterogeneity was observed. Six studies reported the seroprevalence of TORCH agents among pregnant women, with a seroprevalence estimate of 27.7% (95% CI [5.9–70.1]; *I*^2^ = 99.69%; *p* < 0.001) (Table 2). The pooled seroprevalence (from 13 studies) of TORCH agents among infants was 35.1% (95% CI [16.2–60.2]; *I*^2^ = 99.12%; *p* < 0.001) (Table 2). Further, the forest plot for the subgroup meta-analysis by the target population and all other subgroup forest plots are provided as a File S5).

**Table 2:** Subgroup analysis for comparison of TORCH agents among the target population in Malaysia.

**Target population**	**Number of studies**	**Prevalence (%)**	**95% CI**	***I***^2^ (%)	***Q***	**Heterogeneity**	
						**DF**	***p-*** **value**
**Pregnant women**	6	27.7	5.9–70.1	99.69	1,619.536	5	<0.001
**Infants**	13	35.1	16.2–60.2	99.12	1,366.689	12	<0.001
**Overall**	19	32.5	16.6–53.8	99.4	3,021.897	18	<0.001

The outcome of the subgroup meta-analysis based on the type of TORCH agents among pregnant women showed that the highest seroprevalence (93.2%) was from CMV, although the estimate was from a single study. This was followed by *T. gondii* (36.1%) whose estimate was from three studies (Table 3). *T. pallidum* had the lowest seroprevalence (1.1%) among pregnant women. In addition, subgroup meta-analysis of TORCH agents among pregnant women by study location revealed that the majority of studies (*n* = 4) reporting the seroprevalence of TORCH agents in Malaysia were conducted in Kuala Lumpur, with a seroprevalence of 17.4%. A high seroprevalence estimate of 93.2% was derived from a study conducted in multiple locations.

**Table 3:** Subgroup analysis of the prevalence of TORCH infection among pregnant women.

**Subgroup**	**Number of studies**	**Prevalence (%)**	**95% CI**	***I***^2^ (%)	***Q***	**Heterogeneity**	
						**DF**	***p-*** **value**
**TORCH agents**							
*T. gondii*	3	36.1	30.4–42.3	71.7	7.067	2	0.029
*Rubella virus*	1	11.1	8.5–14.3	–	–	–	–
*T. pallidum*	1	1.1	0.9–1.2	–	–	–	–
CMV	1	93.2	88.2–96.2	–	–	–	–
**Locations**							
Kuala Lumpur	4	17.4	2.1–67.6	99.78	1,378.554	3	<0.001
Selangor	1	11.1	8.5–14.3	–	–	–	–
Multilocation	1	93.2	88.2–96.2	–	–	–	–
**Detection method**							
ELISA	4	54.3	33.7–73.5	97.22	107.961	3	<0.001
MEIA	1	11.1	8.5–14.3	–	–	–	–
VDRL & TPHA	1	1.1	0.9–1.2	–	–	–	–

**Notes.**

ELISA Enzyme-linked immunosorbent Assay; MEIA Microparticle Enzymes Immunoassay VDRL Venereal Disease Research Laboratory TPHA Treponema pallidum Hemagglutination Assay

Finally, the outcome of the subgroup meta-analysis based on the detection technique among pregnant women showed that four studies used ELISA, with a seroprevalence of 54.3% (Table 3), followed by two studies each, one utilizing MEIA, with a seroprevalence of 11.1% and VDRL &TPHA with a seroprevalence of 1.1%.

In the subgroup meta-analysis according to the type of TORCH agents in infants. The highest seroprevalence (95.9%) was for CMV, followed by *T. gondii* (16.6%), Rubella (10%), and HSV (5.6%). *T. pallidum* had a low seroprevalence (4%) and was reported in only one study (Table 4).

**Table 4:** Subgroup analysis of the prevalence of TORCH infection among infants.

**Subgroup**	**Number of studies**	**Prevalence (%)**	**95% CI**	***I***^2^ (%)	***Q***	**Heterogeneity**	
						**DF**	***p-*** **value**
**TORCH agents**							
CMV	4	95.9	16.7–100	99.17	359.647	3	<0.001
*T. pallidum*	1	4	3.2–5.1	–	–	–	–
*Rubella virus*	2	10	1.4–46.7	99.35	154.589	1	<0.001
*T. gondii*	3	16.6	1.0–79.7	99.33	297.864	2	<0.001
HSV	3	5.6	0.1–86.1	98.08	211.580	2	<0.001
**Locations**							
Multilocation	8	15.2	5.3–36.3	99.12	796.599	7	<0.001
Kelantan	5	75.5	26.5–96.3	98.36	244.066	4	<0.001
**Detection method**							
ELISA	2	21.4	5.6–55.3	95.81	23.877	1	<0.001
VDRL	1	4	3.2–5.1	–	–	–	–
HI & HIT	2	10	1.4–46.7	99.35	154.589		<0.001
IFA	1	1.1	0.7–1.8	–	–	–	–
CF	3	49.8	0.4–99.5	96.86	63.655	2	<0.001
ECLIA	4	82.5	19.5–98.9	98.75	239.877	3	<0.001

**Notes.**

ELISA Enzyme-linked immunosorbent Assay VDRL Venereal Disease Research Laboratory HI Hemagglutination Inhibition HIT Hemadsorption Immunosorbent Technique IFA Indirect Fluorescence Antibody CF Complement Fixation ECLIA Electrochemiluminescence

Additionally, the subgroup analysis of TORCH agents by study location indicated that most of the studies (*n* = 8) that reported the seroprevalence of TORCH agents in Malaysia were done in multiple locations (city/state), with a seroprevalence of 15.2%. However, a high seroprevalence of 75.5% was observed in Kelantan (Table 4).

The outcomes of the subgroup meta-analysis according to the detection method of TORCH agent among infants showed that four studies used ECLIA, with a seroprevalence of 82.5%. Three studies each used Complement Fixation (CF), with a seroprevalence of 49.8%, followed by two studies each using ELISA and HI & HIT with a seroprevalence of 21.4% and 10%, respectively, (Table 4).

### Quality assessment results

Based on the Joanna Briggs Institute (JBI) critical appraisal tool, the included studies demonstrated high methodological quality (File S3). Five studies achieved a quality score of 100%, five scored 88.9%, and the remaining studies scored 77.8% and 66.7%. All studies demonstrated appropriate sampling frame, proper data analysis coverage, and valid methods for condition identification as shown in the File S4. Because all studies maintained strong overall methodological integrity, none were excluded based on quality scores.

## Discussion

To the best of the author’s knowledge, this is the first systematic review and meta-analysis study to determine the seroprevalence of TORCH agents amongst pregnant women and infants in Malaysia. This study critically assessed seroprevalence based on a comprehensive evaluation of data/records from scientific articles on the seroprevalence of TORCH agents among pregnant women and infants in Malaysia published till the present (November 2025).

This study analyzed 19 reports on the seroprevalence of infection by TORCH agents in Malaysia. An overall seroprevalence of 32.5% was found. Based on the target population, 27.7% and 35.1% seroprevalence were found in pregnant women and infants, respectively. Among the TORCH agent types, CMV in pregnant women demonstrated the highest seroprevalence (93.2%). This outcome could be due to the higher prevalence of perinatal and postnatal CMV infection caused by reactivated latent infection in pregnant women (Saraswathy et al., 2011). A similar outcome was reported by a study in neighboring Singapore where a CMV seroprevalence of 87% was documented (Wong et al., 2000). In Europe, countries with similar findings includes Turkey (84.5%) (Yılmazer et al., 2004), and Spain (84%) (Estripeaut et al., 2007). However, it is worth mentioning that some countries such as Ireland and Australia reported lower seroprevalence of CMV (30.4% (Knowles et al., 2005) and 56.8% (Munro et al., 2005 respectively) among pregnant women, and this could be attributed to the advancement in perinatal and antenatal care to cover TORCH infection. Conversely, an extremely higher seroprevalence were recorded in Thailand (100%) (Taechowisan et al., 1997; Bhattarakosol, Sithidajporn & Bhattarakosol, 1998), and among Chinese pregnant women (98.11%) (Huang et al., 2021).

According to the seroprevalence of TORCH agent types among infants, CMV infection was the most prevalent, at 95.9%. This high seroprevalence points to a high risk of congenital infection due to maternal reinfection and reactivation. Hence, neonatal CMV screening is advocated or should be considered in peri- and antenatal clinics (Wilson et al., 2015). Unfortunately, in most countries, including Malaysia, screening for CMV infection in pregnant women and neonates is not performed routinely and is only demanded when CMV infection is suspected (Ross, Novak & Boppana, 2011; Hamid et al., 2022).

However, when interpreting these infant prevalence rates, the age at the time of testing must be carefully considered. Differentiating between true congenital infections and postnatal transmission relies heavily on this timeframe. For example, true congenital CMV is generally confirmed by polymerase chain reaction (PCR) positivity in blood, urine, or saliva within the first three weeks of life (Leruez-Ville et al., 2024; Baker & Wang, 2025; Demey & Gantt, 2025). Because several of the included studies evaluated older infants or reported aggregated age data (Baker & Wang, 2025), it is highly likely that a portion of the positive infant diagnoses in this meta-analysis represents postnatal transmission or the temporary persistence of maternal antibodies, rather than true congenital infections.

Also, this study revealed that *T. gondii* was the second most common TORCH agent among pregnant women, with a seroprevalence of 36.1%. This seroprevalence rate might be related to the fact that many Malaysians have cats as pets, and that a significant number of them stay in close proximity to wandering cats in their region (Chemoh et al., 2019). The significance of this feline-human transmission pathway is firmly rooted in the foundational discoveries by Dubey, who definitively elucidated the life cycle of *T. gondii* and the environmental risks posed by feline oocyst shedding (Dubey, 2008).

Although the seroprevalence of *T. gondii* varies country-wise, our study outcome was similar to reports from various epidemiological studies of toxoplasmosis seroprevalence among European pregnant women, as seroprevalence of 31.4% in Croatia (Punda-Polić, Tonkić & Capkun, 2000), 33% in Serbia (Bobić et al., 2007), 46% in Greece (Antoniou et al., 2007), 48.6% in Albania (Maggi et al., 2009), 21.5% in Italy (De Paschale et al., 2008), and 28.3% in Thailand (Nissapatorn et al., 2011) have been documented. Surprisingly, a lower rate was reported in certain studies conducted in neighboring Asian nations (3.7% in South Korea (Han et al., 2008), 10.6% in China (Liu et al., 2009), and 17.2% in Singapore (Wong et al., 2000). The low seroprevalence may be attributable to some factors. For example, in the Korean population, the consumption of undercooked or raw meat by humans is limited, domesticated cats are uncommon, the majority of people live in apartments that are not directly associated with soil, water service facilities are well-established, and the majority of people, especially city dwellers, are highly educated (Han et al., 2008).

In accordance with the subgroup meta-analysis of TORCH agent types among infants, the seroprevalence of *T. gondii* was 16.6%. The incidence of congenital Toxoplasma infection in infants is highly associated with their seropositive mothers.

Furthermore, the seroprevalence of Rubella virus among pregnant women was 11.1%, which is comparatively high in comparison to other countries in the Asia-Pacific region, such as Australia (2.7%) (Nardone et al., 2008) and Japan (6.7%) (Okuda et al., 2008), but lower than Singapore (15.8%) (Ang et al., 2010), Thailand (18.0%) (Boonruang & Buppasiri, 2005), Taiwan (16.7%) (Wang et al., 2007), and Sri Lanka (24%) (Palihawadana, Wickremasinghe & Perera, 2003). This finding could be influenced by the age distribution of the included pregnant women. For instance, data from earlier Malaysian cohorts (Ai & Ee, 2008) showed that a significant portion of pregnant women fell into age groups like 21 to 30 years old. This highlights how different age groups and birth cohorts can miss routine childhood vaccination programs entirely. However, more rubella studies are needed to better understand the potential impact of age.

The seroprevalence of the rubella virus among Malaysian infants was 10%. Since 2002, rubella immunization has been replaced with the 2-dose measles-mumps-rubella (MMR) vaccine for all children aged 1 to 7 years. Importantly, this measure or approach for the prevention of rubella susceptibility among pregnant women is still seriously upheld by the government through the Ministry of Health (Cheong, Tong & Khoo, 2013). However, Malaysia still reports cases of rubella infection (Kumar et al., 2023), potentially due to vaccine protection waning over time or the presence of unvaccinated migrant populations. Consequently, a vulnerable population remains at risk of congenital rubella syndrome, underscoring the need for public health authorities to consider strategies like pre-pregnancy screening and booster doses for women of reproductive age.

Infant herpes infection is uncommon, and both HSV-1 and HSV-2 can cause neonatal HSV infection. It varies globally due to several factors, including birth rates and the mother’s HSV seroprevalence status (Samies & James, 2020). Although a low prevalence of 5.6% was reported in this study, the result is similar to other published reports. A rate of 3.6–4% was reported in a study conducted in Tanzania (Saajan et al., 2017), and 5% in Italy (Anzivino et al., 2009). The risk for infant HSV can heighten when a mother is infected with HSV for the first time in late pregnancy. Also, older age and multiparity may be other contributory factors (De Rose et al., 2023).

Among the TORCH agents investigated in this study, *T. pallidum* seroprevalence was the lowest (1.1%) among pregnant women in Malaysia (Goh & Ngeow, 1989), aligning with broader trends in Southeast Asia, where maternal *T. pallidum* incidence decreased from 0.32% in 2012 to 0.21% in 2016. This reduction signifies advancement in tackling this public health concern throughout the region (Korenromp et al., 2019).

This study also found a *T. pallidum* seroprevalence of 4% among infants. The prevalence of congenital *T. pallidum* is reduced in Malaysia and other advanced nations as a result of comprehensive national prenatal screening programs. Antibiotics are administered to pregnant women infected with syphilis in order to treat and prevent further morbidity (Pg Mohammad Hussein et al., 2021). Congenital *T. pallidum* rates are down dramatically since 2012 to 5.0 and 4.0 per 100,000 live births in 2015 and 2016, respectively. According to the WHO, Malaysia was deemed to have eliminated maternal-to-child syphilis transmission in October 2018 (Abd Aziz, 2019).

Furthermore, this study showed that the serological evaluation of TORCH infection in Malaysia is carried out utilizing diverse techniques, with enzyme-linked immunosorbent assay (ELISA) being the predominant detection method, especially in studies investigating pregnant women. In the context of infants, electrochemiluminescence immunoassay (ECLIA) emerged as a relatively recent diagnostic technique, with a high prevalence rate of 82.5%. Both ELISA and ECLIA are the latest detection techniques employed for TORCH infection. Nevertheless, they exhibit disparities in terms of precision. ECLIA generally exhibits greater specificity and improved positive predictive value in comparison to ELISA (Chang et al., 2020). The CF test also accounted for a high seroprevalence among infants (49.8%), but this technique is no longer used for the diagnosis of TORCH infection, and has largely been replaced by more modern techniques like enzyme immunoassay (Batra, Batra & Singh, 2020; Jean-Pierre et al., 2022)

Based on the geographical location, the Kelantan region had the highest TORCH prevalence (75.5%) among infants. Most of the recent TORCH infection studies conducted in Kelantan used a novel method of analyzing paired samples. Implementing serodiagnosis of paired samples helps to decrease the high seroprevalence by enabling precise diagnosis and categorization of patients, which is essential for effective therapy. Moreover, this method can mitigate severe health outcomes in newborns (Thangarajah et al., 2019; Hamid et al., 2022).

The variations in TORCH seroprevalence between Malaysia and neighboring nations reflect distinct socioeconomic, environmental, and healthcare factors (Fuchs et al., 2021). The high CMV rates across the region highlight the persistent, unavoidable circulation of the virus in tropical climates, whereas lower rates in highly urbanized nations point to the protective effects of advanced sanitation and lower household density (Fowler et al., 2022). Similarly, *T. gondii* exposure is heavily driven by environmental contact with stray feline populations and localized cultural dietary habits unique to domestic settings across different countries (Singh, 2003; Dubey, 2008).

These trends have critical translational implications for Malaysian healthcare policies. First, while the national rubella immunization program has been highly effective, persistent infant cases indicate localized immunity gaps, highlighting a need for targeted MMR catch-up campaigns for migrating workforces from endemic regions (Cheong, Tong & Khoo, 2013). Second, the heavy infant CMV burden provides a strong justification to transition from a reactive “suspected-case only” screening model to a proactive, targeted strategy, such as automatically triggering newborn CMV testing following a failed universal newborn hearing screening (Hamid et al., 2022). Finally, establishing a centralized national TORCH surveillance registry is crucial. This will provide policymakers with the necessary baseline data to conduct formal cost-effectiveness assessments and eventually implement standardized, national prenatal screening guidelines in Malaysia.

This study is limited by the relatively small number of eligible full-text studies (*n* = 13), which may affect the generalizability of the findings. This also resulted in relatively small sample sizes for certain specific TORCH pathogens, and not all pathogens were represented across both the pregnant women and infant population groups. Additionally, there was significant statistical heterogeneity, which likely reflects variations in study populations, geographical locations, and the diverse diagnostic techniques used across different decades.

## Conclusions

The seropositivity of TORCH infection among pregnant women and infants in Malaysia is rather high, with an overall estimated seroprevalence of 32.5%. However, due to limited published data within Malaysia, further seroprevalence studies on TORCH infection are necessary to further assess and evaluate the TORCH agent’s rising incidence in pregnant women and infants in Malaysia.

## Supplemental Information

10.7717/peerj.21632/supp-1 Supplemental Information 1 PRISMA checklist

10.7717/peerj.21632/supp-2 Supplemental Information 2 Search strategy

10.7717/peerj.21632/supp-3 Supplemental Information 3 Critical Appraisal tools for use in JBI Systematic Reviews

10.7717/peerj.21632/supp-4 Supplemental Information 4 Quality assessment of the included studies

10.7717/peerj.21632/supp-5 Supplemental Information 5 Forest plots for subgroup analyses

10.7717/peerj.21632/supp-6 Supplemental Information 6 Systematic Review and/or Meta-Analysis Rationale

## Additional Information and Declarations

### Data Availability

The following information was supplied regarding data availability: This is a Systematic review/Meta-analysis.
