# Supplementary material for: Seroprevalence of toxoplasmosis, rubella, cytomegalovirus, herpes simplex, and syphilis infections among pregnant women and infants in Malaysia: a systematic review and meta-analysis
Source: PeerJ. 2026 Aug 18;14:e21632. doi: 10.7717/peerj.21632 (PMC13499533; doi:10.7717/peerj.21632)
Supplement: Supplementary file 2 [file peerj-14-21632-s002.docx]

**File s1: Search strategy** **2025 November 28 total is** **891**

| **PubMed** | (((((((((((Toxoplasma[Title/Abstract]) OR (toxoplasmosis[Title/Abstract])) OR (Syphilis[Title/Abstract])) OR (“Rubella virus”[Title/Abstract])) OR (Rubella[Title/Abstract])) OR (Cytomegalovirus[Title/Abstract])) OR (CMV[Title/Abstract])) OR (“Herpes simplex virus”[Title/Abstract])) OR (HSV[Title/Abstract])) OR (HSV-1[Title/Abstract])) OR (HSV-2[Title/Abstract])) AND ("Malaysia"[Title/Abstract]) | 141 |
| --- | --- | --- |
| **Scopus** | TITLE-ABS(Toxoplasma OR Toxoplasmosis OR Syphilis OR "Rubella virus" OR Rubella OR Cytomegalovirus OR CMV OR "Herpes simplex virus" OR HSV OR HSV-1 OR HSV-2) AND TITLE-ABS("Malaysia") | 556 |
| **Google Scholar** | allintitle:(Toxoplasma OR Toxoplasma OR Syphilis OR “Rubella virus” OR Rubella OR Cytomegalovirus OR CMV OR “Herpes simplex virus” OR HSV OR HSV-1 OR HSV-2) ("Malaysia") | 104 |
| **Web Science title** | (((((((((((TI=(Toxoplasma)) OR TI=(toxoplasmosis)) OR TI=(syphilis)) OR TI=(Rubella)) OR TI=(“Rubella Virus”)) OR TI=(Cytomegalovirus)) OR TI=(CMV)) OR TI=(“Herpes simplex virus”)) OR TI=(HSV)) OR TI=(HSV-1)) OR TI=(HSV-2)) AND TI=("Malaysia") | 44 |
| **Web Science abstract** | (((((((((((AB=(Toxoplasma)) OR AB=(toxoplasmosis)) OR AB=(syphilis)) AND AB=(Rubella)) OR TI=(“Rubella Virus”)) OR AB=(cytomegalovirus)) OR AB=(CMV)) OR AB=(Herpes simplex virus)) OR AB=(HSV)) OR AB=(HSV-1)) OR AB=(HSV-2)) AND AB=("Malaysia") | 46 |
