# Supplementary material for: Seroprevalence of toxoplasmosis, rubella, cytomegalovirus, herpes simplex, and syphilis infections among pregnant women and infants in Malaysia: a systematic review and meta-analysis
Source: PeerJ. 2026 Aug 18;14:e21632. doi: 10.7717/peerj.21632 (PMC13499533; doi:10.7717/peerj.21632)
Supplement: Supplementary file 4 [file peerj-14-21632-s004.docx]

| **File S3: Table 2**. Quality assessment of the included studies. | | | | | | | | | | | |
| --- | --- | --- | --- | --- | --- | --- | --- | --- | --- | --- | --- |
| **No.** | **Study ID** | **Questions assessing included studies** | | | | | | | | | **Yes (%)** |
|  |  | **1** | **2** | **3** | **4** | **5** | **6** | **7** | **8** | **9** |  |
| 1 | Ahmad et al. | Y | Y | Y | Y | Y | Y | Y | Y | Y | 100 |
| 2 | Ai. | Y | Y | Y | Y | Y | Y | Y | Y | U | 88.9 |
| 3 | Andiappan et al. | Y | Y | Y | Y | Y | Y | Y | Y | Y | 100 |
| 4 | Balasubramaniam et al. a | Y | U | Y | U | Y | Y | Y | N | Y | 77.8 |
| 5 | Elmi et al. | Y | Y | Y | Y | Y | Y | Y | Y | Y | 100 |
| 6 | Elmi et al. 2 | Y | Y | Y | Y | Y | Y | Y | Y | Y | 100 |
| 7 | Emelia et al. | Y | Y | Y | Y | Y | Y | Y | Y | Y | 100 |
| 8 | Goh. | Y | Y | Y | Y | Y | Y | Y | U | Y | 88.9 |
| 9 | Hamid et al. | Y | Y | Y | Y | Y | Y | Y | Y | U | 88.9 |
| 10 | Mohamed | Y | Y | N | Y | Y | Y | Y | Y | Y | 88.9 |
| 11 | Saraswathy et al. | Y | Y | Y | Y | Y | Y | Y | Y | U | 88.9 |
| 12 | Tan. | Y | Y | Y | Y | Y | Y | Y | Y | Y | 100 |
| 13 | Tan et al. a | Y | Y | Y | N | Y | Y | U | Y | U | 66.7 |
|  | | | | | | | | | | | |

1. Was the sample frame appropriate to address the target population? 2. Were study participants sampled in an appropriate way? 3. Was the sample size adequate? 4. Were the study subjects and the setting described in detail? 5. Was the data analysis conducted with sufficient coverage of the identified sample? 6. Were valid methods used for the identification of the condition? 7. Was the condition measured in a standard, reliable way for all participants? 8. Was there appropriate statistical analysis? 9. Was the response rate adequate, and if not, was the low response rate managed appropriately? Y=Yes; N=No; U=Unclear or NA Not applicable
