# Supplementary material for: Seroprevalence of toxoplasmosis, rubella, cytomegalovirus, herpes simplex, and syphilis infections among pregnant women and infants in Malaysia: a systematic review and meta-analysis
Source: PeerJ. 2026 Aug 18;14:e21632. doi: 10.7717/peerj.21632 (PMC13499533; doi:10.7717/peerj.21632)
Supplement: Supplementary file 5 [file peerj-14-21632-s005.docx]

**File s4: Forest plots for subgroup analyses**

**Figure s4a.** Forest plot showing the subgroup meta-analysis comparison of TORCH agents among the target population in Malaysia.

**Figure s4b.** Subgroup meta-analysis of causative TORCH agents in pregnant women in Malaysia.

**Figure s4c.** Forest plot of subgroup meta-analysis by type of TORCH agents in Malaysian infants.

**Figure s4d.** Forest plot of subgroup meta-analysis by study location among pregnant women.

**Figure s4e.** Forest plot of infant subgroup meta-analysis by study location.

**Figure s4f.** Forest plot of subgroup meta-analysis by method of detection of TORCH agents among pregnant women.

**Figure s4g.** Forest plot of subgroup meta-analysis by method of detection of TORCH agents among infants.
