# Supplementary material for: Seroprevalence of toxoplasmosis, rubella, cytomegalovirus, herpes simplex, and syphilis infections among pregnant women and infants in Malaysia: a systematic review and meta-analysis
Source: PeerJ. 2026 Aug 18;14:e21632. doi: 10.7717/peerj.21632 (PMC13499533; doi:10.7717/peerj.21632)
Supplement: Supplementary file 6 [file peerj-14-21632-s006.docx]

**Systematic Review and/or Meta-Analysis Rationale**

This study addresses the absence of a comprehensive and reliable dataset for TORCH infections in Malaysia. Although Malaysia seeks to improve maternal health, the lack of routine, nationwide prenatal screening and the fragmentation of current data restricted to localized areas or individual infections, results in a considerable knowledge gap. This systematic review presents the first comprehensive meta-analysis to inform public health policy by providing a unified, robust dataset. The results are primarily aimed at public health officials and policymakers in Malaysia to inform national prenatal screening strategies. Additionally, the study aims to assist obstetricians, pediatricians, and clinical microbiologists in Southeast Asia region and beyond in enhancing early detection and intervention for pregnant women and newborns susceptible to vertical transmission. Because recent studies reported extremely high seropositivity, such as 95.9% for CMV in infants, this review is necessary to help clinicians differentiate between acute infection and passive immunity from the mother, which remains a primary diagnostic challenge.
